# Research on the Dual Modulation of All-Fiber Optic Current Sensor

**DOI:** 10.3390/s22020430

**Published:** 2022-01-07

**Authors:** Jianhua Wu, Xiaofeng Zhang, Liang Chen

**Affiliations:** College of Electrical Engineering, Naval University of Engineering, Wuhan 430033, China; jianhuafly@163.com (J.W.); zhangxiaofeng201@126.com (X.Z.)

**Keywords:** all-fiber optic current sensor, dual modulation, correlation demodulation algorithm, frequency multiplication factor

## Abstract

Acousto-optic modulator (AOM) and electro-optical modulator (EOM) are applied to realize the all-fiber current sensor with a pulsed light source. The pulsed light is realized by amplitude modulation with AOM. The reflected interferometer current sensor is constructed by the mirror and phase modulation with EOM to improve the anti-interference ability. A correlation demodulation algorithm is applied for data processing. The influence of the modulation frequency and duty cycle of AOM on the optical system is determined by modeling and experiment. The duty cycle is the main factor affecting the normalized scale factor of the system. The modulation frequency mainly affects the output amplitude of the correlation demodulation and the system signal-to-noise ratio. The frequency multiplication factor links AOM and EOM, primarily affecting the ratio error. When the frequency multiplication factor is equal to the duty cycle of AOM and it is an integer multiple of 0.1, the ratio error of the system is less than 1.8% and the sensitivity and the resolution of AFOCS are 0.01063 mV/mA and 3 mA, respectively. The measurement range of AFOCS is from 11 mA to 196.62 A, which is excellent enough to meet the practical requirements for microcurrent measurement.

## 1. Introduction

The all-fiber optic current sensor (AFOCS) can measure the current accurately based on the Faraday effect and Ampere circuital theorem [1]. AFOCS has many advantages, such as excellent insulation characteristics, simultaneous measurement of AC and DC, flexible sensor diameter, and digital output [2]. It has gradually replaced electromagnetic current transformers in the fields of pulsed current measurement [3], ultra-high voltage electrical network [4], and plasma current measurement [5]. The AFOCS is essential and will play a critical role in the smart grid [6].

Learning from the fiber optic gyroscope principle, the AFOCS also uses a continuous wide spectrum light source to reduce the coherence length, restrict the influence of backscattered light, and improve the signal-to-noise signal ratio of the system [7]. However, some applications need a pulsed light source, such as to suppress the backscattered light [8], suppress power frequency interference in AC measurement applications [9], and the weak current measurement with the fiber loop architecture [10]. Therefore, research of AFOCS with pulsed light sources has essentially practical significance. Currently, there are two types of pulsed light sources. One is the laser diodes. Zhang et al. use laser diodes as pulsed light sources to study the loop structure with single-polarization single-mode couplers [11]. The other type is the pulsed light source generated by an acousto-optic modulator (AOM). Du et al. use AOM for pulse regulation when studying the sensitivity of all-fiber optic current with recirculating loop structure [12]. These methods both adopt the polarimetric current sensor. However, the optical system of the polarimetric current sensor has the disadvantage of the weak anti-interference ability, which is an enormous challenge in practical engineering. 

In this paper, an optical interferometer current sensor with pulsed light is constructed to suppress external interference and restrict the influence of backscattered light. An electro-optical modulator (EOM) is applied to phase modulation. AOM is used to generate the pulsed light source simultaneously. Under the dual modulation of AOM and EOM, AFOCS with pulsed light is conducted. This article mainly talks about the relationship of modulation signal between AOM and EOM. In a particular case, when the frequency multiplication factor is equal to the duty cycle of AOM while the frequency multiplication factor is an integer multiple of 0.1, the sensitivity and the resolution of AFOCS are 0.01063 mV/mA and 3 mA, respectively. The ratio error is less than 0.3%, which can meet the requirement of microcurrent measurement applications, such as partial discharge detection [13] and leakage detection [14]. 

The remainder of the paper is organized as follows. Section 2 describes the principle of operation. The optical design and data processing method are also discussed in this section. In Section 3, we model and simulate to explore the influence of modulation parameters on AFOCS. An experiment is established to verify the correctness of the theory in Section 4. The conclusion is in Section 5.

## 2. Principle of Operation

### 2.1. Optical Design

**Figure 1:**
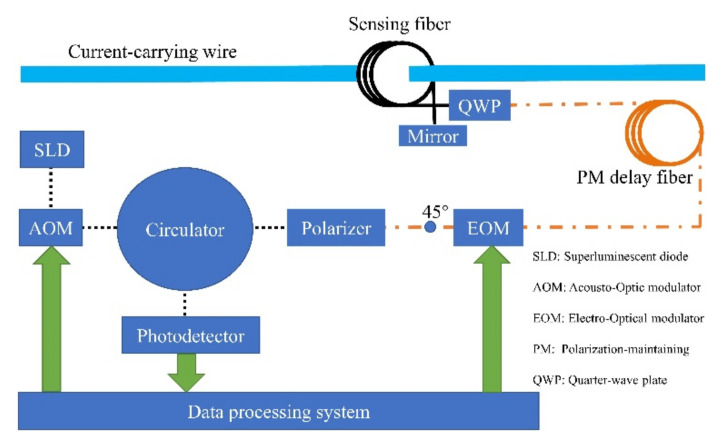
The structure of AFOCS.

The polarization state of the polarized light is adjusted by the magnetic field generated by the current to be measured. The rotation angle of the polarization plane is proportional to the electric current based on the principle of the Faraday effect [15]. AOM is applied to generate a pulsed light source based on a reflective interferometer current sensor. The structure of AFOCS is shown in Figure 1.

The black dotted line represents single-mode optical fiber, which mainly realizes energy transmission. The orange dot-dash line represents the polarization-maintaining fiber, which can reduce the influence of environmental factors on the polarization state. The spun highly birefringent fiber is applied as the sensing fiber, which is shown as the solid black line in Figure 1. The green arrow line represents the electrical signal transmission, including photoelectric signal conversion, modulation signal generation, and data processing. The current-carrying wire is the light blue line, as shown in Figure 1. 

The principle is as follows. Continuous light of the superluminescent diode will be modulated to pulsed light by AOM. The frequency of pulsed light is adjustable, and the amplitude of pulsed light is the same as continuous light. After passing through the circulator, the pulsed light is sent to the polarizer, and then it will change into linearly polarized light. The polarized light will convert into two linearly polarized light beams when the optic axis of the polarizer and EOM is aligned with a 45° offset.Therefore, the input polarized light will be equally launched into both polarization modes. These two linearly polarized lights are orthogonal and convert into left-handed and right-handed circularly polarized light after passing through the quarter-wave plate. The phase velocities of the two circularly polarized light beams are different when they pass through the magnetic field generated by the electric current to be measured. The Faraday rotation angle is applied to represent the rotation angle of the polarization plane. The Faraday rotation angle is doubled when the circularly polarized light beams come back in the same optical path when the mirror reflects them. The circularly polarized lights convert into linearly polarized light when they pass through the quarter-wave plate. Finally, the interference occurs at the polarizer. The light intensity with electric current information is sent to the photodetector through the circulator and processed by the data processing system. EOM is used to modulate the phase and improve the sensitivity of AFOCS. The modulation frequency is related to the optical path length, which is adjusted by the polarization-maintaining delay fiber and sensing fiber [16]. When the action of AOM is not considered, the light power detected by the photodetector is [1]

(1) $$P(t)=P_{0}\cdot \frac{1+\cos[\theta -\Delta \varphi (t)]}{2}$$

Here, $P_{0}$ is proportional to the source power. Δφ denotes the phase difference modulated by EOM and τ represents the transmission delay in the fiber. φ(t) is the modulated signal in the forward transmitting direction, φ(t+τ) expresses the modulated signal in the backward transmitting direction with τ time delay. θ=4NVI denotes the Faraday rotation angle, N is the number of turns of the sensing fiber coil wrapped around the current-carrying wire. V represents the Verdet constant related to the material of sensing fiber, the working wavelength of the light source, and the operating temperature [17]. I expresses the electric current to be measured. 

The modulation signal of AOM is a periodic gate signal.

(2) $$x(t)=\left\{\begin{array}{l} 1\begin{array}{ccc} & & nt_{d}+t\leq t_{0}+nt_{d} \end{array}\\ 0\begin{array}{ccc} & & others \end{array} \end{array}\right.$$

Here, t≥0. $t_{d}$ and $t_{0}$ represent the period of the modulation signal and the pulse width, respectively. The duty cycle of the modulation signal is $RD=t_{0}/t_{d}$. *n* denotes a positive integer, which expresses the number of pulse repetition periods. After amplitude modulation by AOM, the detected optical power is

(3) $$P^{\prime }(t)=\left\{\begin{array}{l} P_{0}\cdot \frac{1+\cos[\theta -\Delta \varphi (t)]}{2}\begin{array}{ccc} & & nt_{d}+t\leq t_{0}+nt_{d} \end{array}\\ 0\begin{array}{ccc} & & others \end{array} \end{array}\right.$$

The door signal can be converted into a periodic square wave signal.

(4) $$x(t)=\frac{f(t)+1}{2}$$

Here, f(t) represents a periodic square wave signal.

(5) $$f(t)=\left\{\begin{array}{l} 1\begin{array}{ccc} & & nt_{d}+t\leq t_{0}+nt_{d} \end{array}\\ -1\begin{array}{ccc} & & t_{0}+nt_{d}\leq nt_{d}+t\leq (n+1)t_{d} \end{array} \end{array}\right.$$

Taking Equations (4) and (5) into Equation (3), and the detected optical power is

(6) $$P^{\prime }(t)=P_{0}\cdot \frac{1+\cos[\theta -\Delta \varphi (t)]}{2}\cdot \frac{1+f(t)}{2}=A(t)+B(t)$$

Here, $A(t)=P_{0}\cdot \{1+\cos[\theta -\Delta \varphi (t)]\}/4$ represents the detected optical power when the light source is continuous, and $B(t)=P_{0}\cdot \{1+\cos[\theta -\Delta \varphi (t)]\}\cdot f(t)/4$ denotes the optical power when the light source is modulated by the square signal.

**Figure 2:**
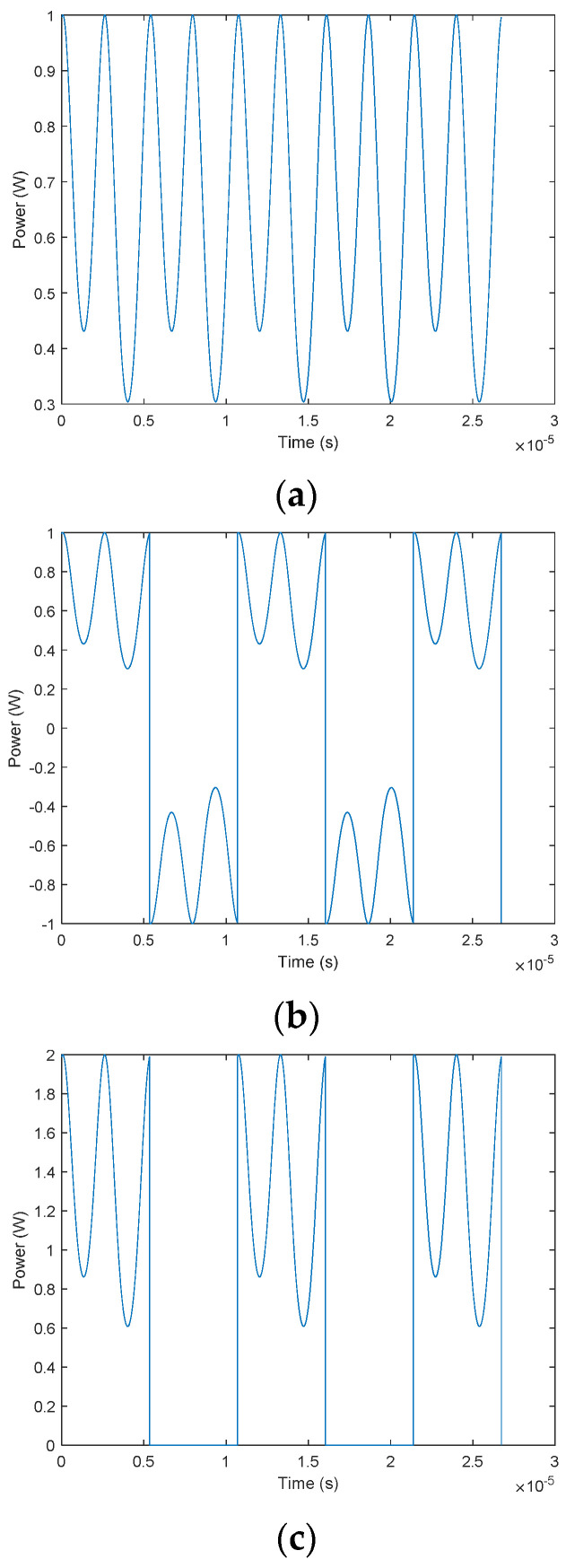
The light power detected by the photodetector. (**a**) The light power curve of *A*(*t*). (**b**) The light power curve of *B*(*t*). (**c**) The light power curve of the sum of *A*(*t*) and *B*(*t*).

The Faraday rotation angle is 0.132 rad, the modulation signal of AOM is a square wave signal, the duty cycle is 50%, and the modulation frequency and amplitude of AOM are 93.5 kHz and 1 V, respectively. EOM adopts sine wave modulation. The modulation frequency and amplitude of EOM are 187 kHz and 0.9205 V, respectively. MATLAB carries out the simulations are shown in Figure 2.

The power detected by the photodetector is the periodic modulation signal when the light source is continuous, as shown in Figure 2a. When the square signal is applied for light source amplitude modulation, the detected light power is periodically truncated and flipped compared with continuous light-output power, as shown in Figure 2b. Finally, the output power is the sum of the previous cases, as shown in Figure 2c.

### 2.2. Signal Demodulation Algorithm

The periodic square wave signal is expressed by the Fourier series [18].

(7) $$f(t)=2RD-1+\frac{4}{\pi }\sum_{m=1}^{\infty }\left[\frac{1}{m}\cdot \sin(m\pi RD\right)\cdot \cos(m\pi RD-m\omega t)]$$

Here, $\omega =2\pi /t_{d}$ is the angular frequency of the square wave modulated by AOM. $t_{d}$, RD, and t are defined as Equation (2). 

EOM adopts a sine wave as the modulation signal. The modulation signal of EOM is $\phi (t)=A\sin\omega_{0}t$. A and $\omega_{0}$ represent the amplitude and angular frequency of the modulation signal. Then,

(8) $$\Delta \phi =\phi (t+\tau )-\phi (t)=2A\sin\frac{\omega_{0}\tau }{2}\cdot \cos(\omega_{0}t+\frac{\omega_{0}\tau }{2})$$

When $\omega_{0}\tau =\pi$ rad and δ=2A, Equation (8) can be simplified as

(9) $$\Delta \phi =-\delta \cdot \sin\omega_{0}t$$

The trigonometric function and the first kind Bessel function [19] are applied in Equation (6), and the light power detected by the photodetector is given by

(10) $$\begin{array}{l} P^{\prime }(t)=\frac{P_{0}}{4}\{1+\cos\theta \cdot [J_{0}(\delta )+2\sum_{n=1}^{\infty }J_{2n}(\delta )\cdot \cos(2n\omega_{0}t)]-\sin\theta \cdot 2\sum_{n=1}^{\infty }J_{2n-1}(\delta )\cdot \sin[(2n-1)\omega_{0}t]\}\\ \cdot \{2RD+\frac{4}{\pi }\sum_{m=1}^{\infty }\left[\frac{1}{m}\cdot \sin(m\pi RD\right)\cdot \cos(m\pi RD-m\omega t)]\} \end{array}$$

We define an essential parameter as the frequency multiplication factor. The frequency multiplication factor is the ratio of AOM modulation angular frequency to EOM modulation angular frequency.

(11) $$k=\frac{\omega }{\omega_{0}}$$

Here, ω and $\omega_{0}$ represent the angular frequency of AOM and EOM, respectively. k expresses the frequency multiplication factor. 

The detected power mainly concentrates on low-order harmonic components, so we only consider the harmonic signals n=1. Equation (10) can be simplified as

(12) $$\begin{array}{l} P^{\prime }(t)=\frac{P_{0}}{4}[1+J_{0}(\delta )\cos\theta +2J_{2}(\delta )\cdot \cos(2\omega_{0}t)\cos\theta -\sin\theta \cdot 2J_{1}(\delta )\cdot \sin(\omega_{0}t)]\\ \cdot \{2RD+\frac{4}{\pi }\sum_{m=1}^{\infty }\left[\frac{1}{m}\cdot \sin(m\pi RD\right)\cdot \cos(m\pi RD-mk\omega_{0}t)]\} \end{array}$$

**Figure 3:**
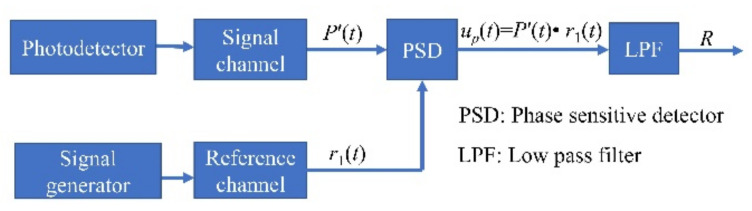
The principle of correlation detection.

The correlation detection method is applied for data demodulation [20]. The principle of correlation detection is shown in Figure 3.

There are two channels in the phase-sensitive detector (PSD): the signal and reference channels. The electric signals from photodetector ($P^{\prime }(t)$) and signal generator ($r_{1}=\sin\omega_{0}t$) enter the signal channel and reference channel, respectively. The output of PSD ($u_{p}(t)$) is the product of these two input signals. A low-pass filter (LPF) is applied to eliminate the difference-frequency and sum-frequency terms.

We define another essential parameter as the duty cycle correlation coefficient. The duty cycle correlation coefficient $m_{0}$ is the ratio of duty cycle to frequency multiplication factor.

(13) $$m_{0}=\frac{RD}{k}$$

Here, *RD* and k denote the duty cycle and the frequency multiplication factor as previously defined, respectively. If $m_{0}$ is a positive integer, which indicates the frequency multiplication factor is related to the duty cycle. Otherwise, the frequency multiplication factor is not related to the duty cycle. The result of the correlation demodulation is

(14) $$R=\left\{\begin{array}{l} -\frac{RD\cdot P_{0}\cdot J_{1}(\delta )\cdot \sin\theta }{2}+\frac{kP_{0}\sin^{2}(\pi m_{0})[J_{0}(\delta )-J_{2}(\delta )]\cos\theta }{2\pi }+\frac{kP_{0}\sin^{2}(\pi m_{0})}{2\pi }\begin{array}{cc} & mk=1 \end{array}\\ -\frac{RD\cdot P_{0}\cdot J_{1}(\delta )\cdot \sin\theta }{2}\begin{array}{cccc} & & & \begin{array}{cc} & \begin{array}{cc} & \begin{array}{c} mk\neq 1 \end{array} \end{array} \end{array} \end{array} \end{array}\right.$$

When mk=1 and the frequency multiplication factor is not related to the duty cycle. The coherent demodulation result of the system is

(15) $$R=-H\sin(\theta -\xi )+H^{\prime }$$

Here, $H^{\prime }=kP_{0}\sin^{2}(\pi RD/k)/(2\pi )$, $H=P_{0}\sqrt{{\left\{J_{1}(\delta )RD/2\right\}}^{2}+{\left\{k[J_{0}(\delta )-J_{2}(\delta )]\sin^{2}(\pi RD/k)/(2\pi )\right\}}^{2}}$, and $\tan\xi =-k\sin^{2}(\pi RD/k)[J_{0}(\delta )-J_{2}(\delta )]/[\pi RD\cdot J_{1}(\delta )]$.

The Faraday rotation angle is

(16) $$\theta =-\arcsin\frac{R-H^{\prime }}{H}+\xi$$

In this case, the Faraday rotation angle is related to the frequency multiplication factor and the duty cycle of AOM.

When mk=1 and the frequency multiplication factor is associated with the duty cycle ($m_{0}$ is a positive integer and $\sin(\pi m_{0})=0$). This case will have the same result as mk≠1, and we analyze them simultaneously as follows. 

The Faraday rotation angle is

(17) $$\theta =-\mathrm{arc}\sin\frac{2R}{RD\cdot P_{0}\cdot J_{1}(\delta )}$$

According to Equation (17), the Faraday rotation angle is only related to the duty cycle of AOM and has nothing to do with the frequency multiplication factor.

## 3. Modeling and Simulation

The ratio error and scale factor are applied as the evaluation criterion of AFOCS.

### 3.1. Ratio Error

The ratio error of the optical system is defined as

(18) $$\eta =\left|\frac{\bar{\theta }-\theta }{\theta }\right|\times 100\%=\left|\frac{\bar{I}-I}{I}\right|\times 100\%$$

Here, $\bar{\theta }$ and $\bar{I}$ are the measured value of the Faraday rotation angle and electric current, respectively. θ and I denote the reference values of the Faraday rotation angle and the electric current, respectively. η represents the ratio error.

**Figure 4:**
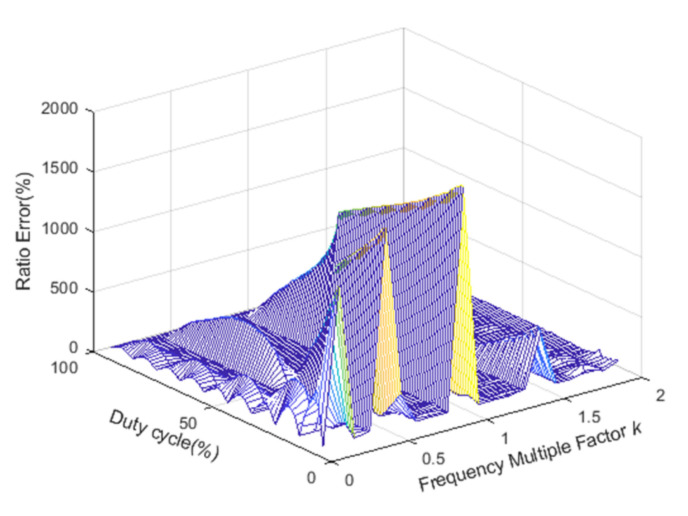
The relationship between the ratio error and the duty cycle or the frequency multiplication factor.

When the duty cycle is from 10% to 99% and the frequency multiplication factor is from 0.1 to 2, the relationship between the ratio error and the duty cycle or the frequency multiplication factor is shown in Figure 4.

The ratio error changes with the duty cycle and the frequency multiplication factor, as shown in Figure 4. However, it is difficult to obtain the mathematical relationship directly from Figure 4. Therefore, we will analyze the duty cycle and frequency multiplication factor separately to get a clear connection with the ratio error.

#### 3.1.1. The Effects of Frequency Multiplication Factor

**Figure 5:**
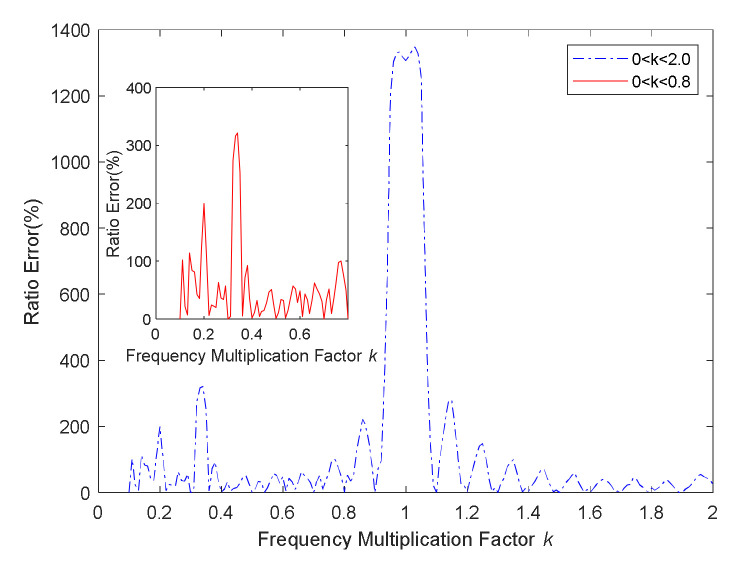
The relationship between ratio error and frequency multiplication factor.

We fix the duty cycle of AOM and simulate the relationship between the ratio error and the frequency multiplication factor. The Faraday rotation angle is θ=0.125 rad. $\bar{\theta }$ is the angle when we compute the faraday rotation angle by Equations (16) and (17). The modulation signal of EOM is a sine wave with a modulation frequency of 50 kHz and an amplitude of 0.9205 V. The modulation signal of AOM is a square wave signal with a duty cycle of 50%. The relationship between ratio error and frequency multiplication factor is shown in Figure 5.

When the modulation frequency of AOM is greater than that of EOM (*k* > 1), the ratio error of the system decreases with fluctuation. When the modulation frequency of AOM is smaller than that of EOM (*k* < 1), the ratio error of the system fluctuates with frequency. When *k* is less than 0.8, a subplot is drawn to describe the relationship between ratio error and frequency multiplication factor within Figure 5 with the red line. There are some peak points where the ratio error is more prominent than 100%. There are also many trough values on the waveform simultaneously, which will be described later.

**Figure 6:**
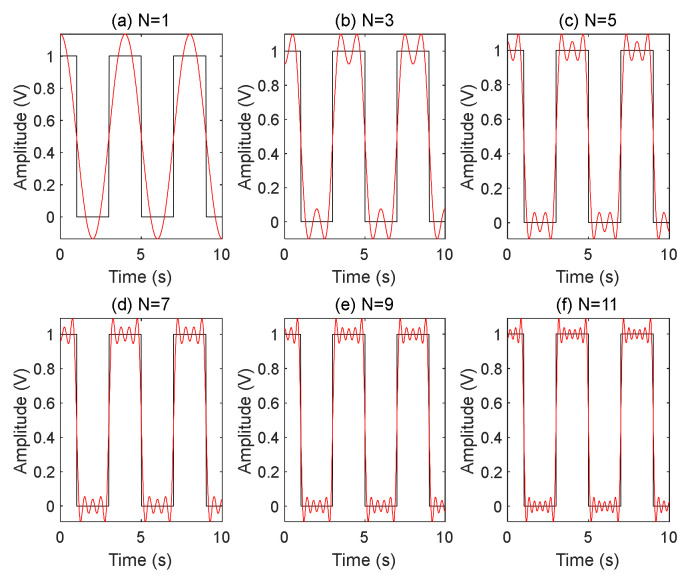
Fourier series expansion fits square wave signals when the Fourier transform order *N* is 1 to 11.

When 0.9 < *k* < 1.1, there is a maximum that AFOCS can not be applied. This phenomenon can be explained as follows. When the Fourier series of the square wave signal is expanded, there is an unavoidable spectrum leakage. The Fourier series expansion of the square wave contains only the first harmonic when the modulation frequency of the AOM is the same as that of EOM. Finally, there is some energy leakage. When the duty cycle is 50%, the relationship between the Fourier series expansion of the periodic signal and the original signal is shown in Figure 6.

The black line is the original square wave signal, and the red line is the fitted signal with the sum of the odd number harmonics, as shown in Figure 6. When the number of harmonics is minor, the square wave signal is quite different from the fitted signal. The matched signal tends to be a square wave signal as the Fourier series increases. 

**Figure 7:**
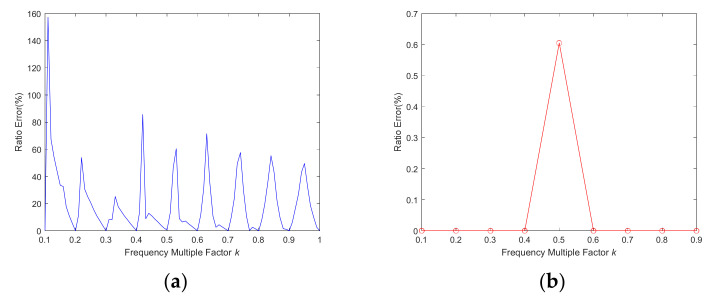
Relationship between the ratio error and frequency multiplication factor. (**a**) Interval = 0.01; (**b**) Interval = 0.1.

The frequency multiplication factor equals the duty cycle as a particular case. The relationship between ratio error and frequency multiplication factor is shown in Figure 7.

When the frequency multiplication factor is equal to the duty cycle of AOM, some troughs will appear periodically, as shown in Figure 7a. When the frequency multiplication factor is an integer multiple of 0.1, the system ratio error will be less than 0.1%, except the frequency multiplication factor equals 0.5, as shown in Figure 7b, which can also be explained as previously with the energy leakage.

#### 3.1.2. The Effects of Duty Cycle

**Figure 8:**
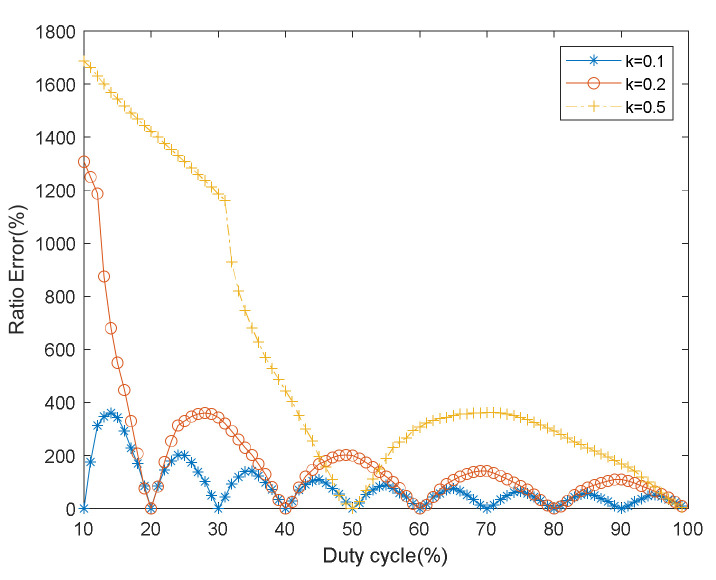
Relationship between the ratio error and duty cycle.

We fix the frequency multiplication factor of AOM and simulate the relationship between the ratio error and duty cycle. The frequency multiplication factor is 0.1, 0.2, and 0.5. The duty cycle is from 10% to 99%, and the interval is 1%. The other parameters of the simulation are the same as Section 3.1.1. The relationship between ratio error and the duty cycle is shown in Figure 8.

The ratio error is minimum when the frequency multiplication factor (except 0.5) is an odd multiple of 0.1, and the duty cycle is integral multiples of 0.1. When the frequency multiplication factor is 0.5, the minimum ratio error will occur when the duty cycle equals 50%. When the frequency multiplication factor is an even multiple of 0.1, and the duty cycle is integral multiples of 0.2, we will also get the minimum ratio error, as shown in Figure 8. Therefore, when the multiplication factor equals duty cycle, and both are the integral multiple of 0.1, the minimum ratio error will be obtained, which is consistent with the above analysis results in Section 3.1.1.

### 3.2. Scale Factor

According to Equations (16) and (17), the Faraday rotation angle is related to the frequency multiplication factor and duty cycle of AOM. The normalized scale factor is

(19) $$\varepsilon =\frac{\lambda (RD,k)}{\max(\lambda (RD,k))}$$

Here, λ(RD,k) represents the scale factor. max(λ(RD,k)) denotes the maximum scale factor when the duty cycle and the frequency multiplication factor are in perfect condition. The output signal of correlation demodulation is

(20) R=ε⋅max(λ(RD,k))⋅θ

**Figure 9:**
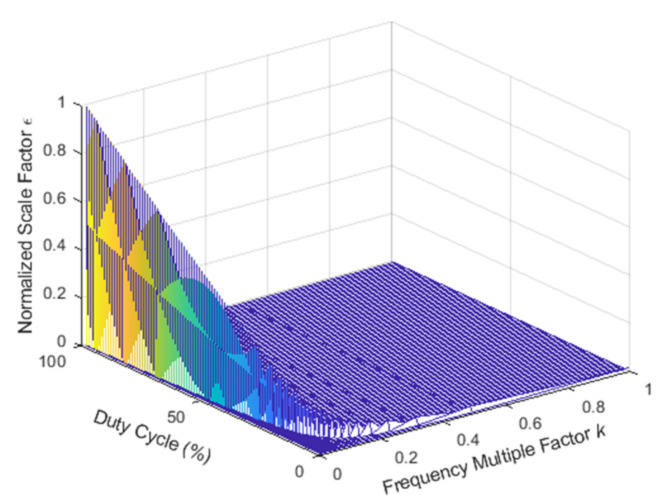
The relationship between the normalized scale factor and the duty cycle or the frequency multiplication factor.

If km=1 and the frequency multiplication factor is not related to the duty cycle, the scale factor is $\lambda (RD,k)=\sqrt{{\left\{J_{1}(\delta )RD/2\right\}}^{2}+{\left\{k[J_{0}(\delta )-J_{2}(\delta )]\sin^{2}(\pi RD/k)/2\pi \right\}}^{2}}$. When the duty cycle is 1% to 99%, the frequency multiplication factor is 1/*m* to 1, and *m* is a positive integer from 2 to 99. The relationship between the normalized scale factor and the duty cycle or the frequency multiplication factor is shown in Figure 9.

**Figure 10:**
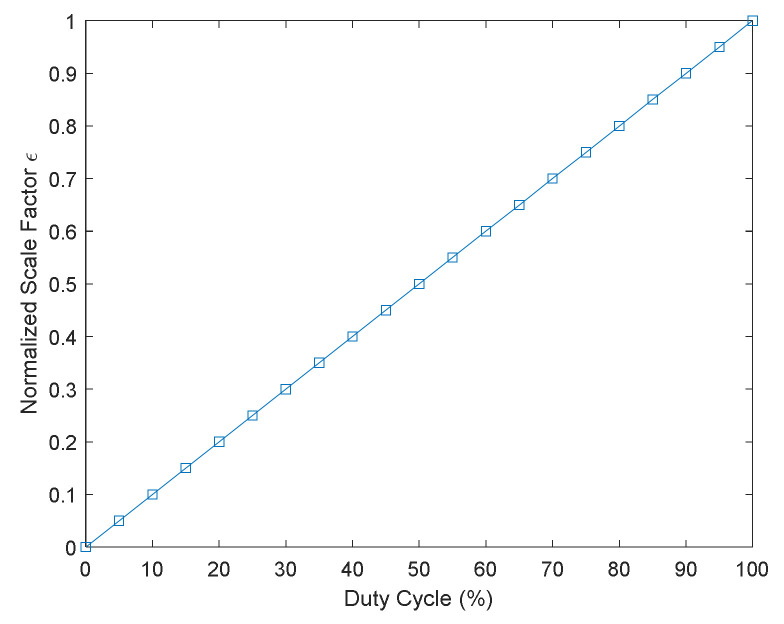
The relationship between the normalized scale factor and the duty cycle.

The duty cycle is the main factor that affects the normalized scale factor, as shown in Figure 9. If km≠1 or km=1, but the frequency multiplication factor is related to the duty cycle, the relationship between the normalized scale factor and the duty cycle is shown in Figure 10.

It can be seen from Figure 10 that the normalized scale factor has a linear relationship with the duty cycle. This phenomenon can be explained by energy. As the duty cycle of AOM increases, the light power detected by the system increases steadily. Therefore, the normalized scale factor rises as the energy received by the detector increases.

Based on Figure 9 and Figure 10, we can conclude that the main factor affecting the normalized scale factor is the duty cycle of AOM, and the frequency multiplication factor is weakly related to the normalized scale factor.

## 4. Experiments and Discussion 

### 4.1. Experimental System 

**Figure 11:**
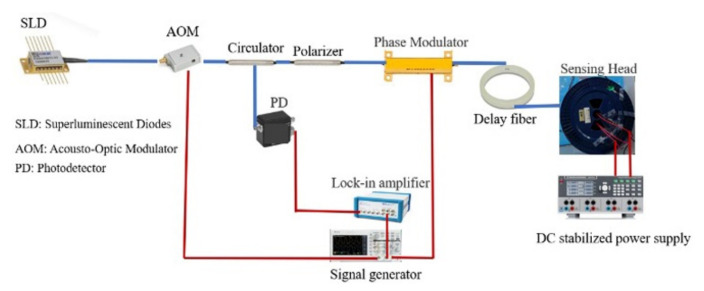
Schematic diagram of the experimental system.

According to the principle shown in Figure 1, the experiment was constructed, as shown in Figure 11.

The blue and red lines in Figure 11 represent the optical and electrical signals, respectively. The sensor head includes a sensing fiber, a mirror, and a quarter-wave plate. The main parameters of experimental equipment are shown in Table 1.

### 4.2. Result and Discussion

#### 4.2.1. The Effect of Duty Cycle

**Figure 12:**
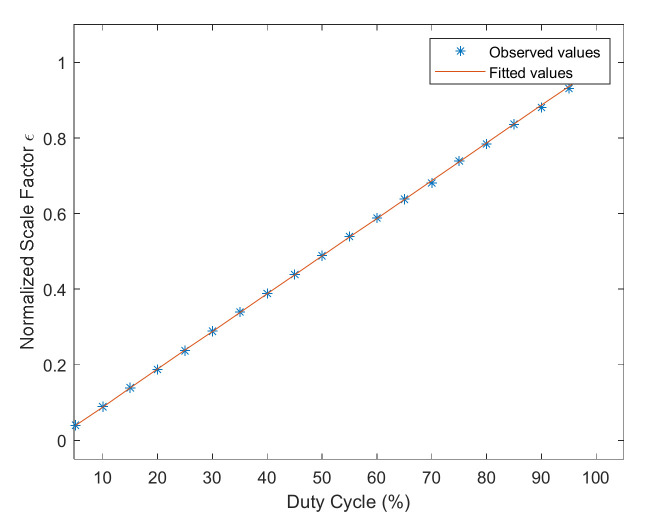
The relationship between normalized scale factor and duty cycle.

We fixed the frequency multiplication factor and tested the normalized scale factor and duty cycle relationships. The duty cycle of the AOM is from 5% to 100%, and the interval is 5%. The relationship between the normalized scale factor and the duty cycle is shown in Figure 12.

The normalized scale factor of the system increases with the duty cycle, which is consistent with the simulation results in Figure 10. The duty cycle of 100% is the continuous light, which has the most significant normalized scale factor.

**Figure 13:**
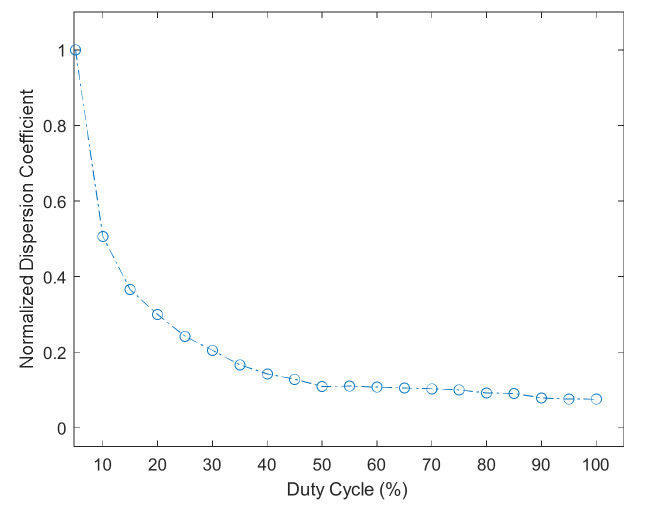
The relationship between normalized dispersion coefficient and duty cycle.

We introduce the dispersion coefficient to characterize the relationship between duty cycle and system anti-interference ability. The dispersion coefficient is defined as the ratio of standard deviation to mean value. The electric current to be measured was 2 A, and we eliminated the influence of zero bias. The dispersion coefficient of the Faraday rotation angle was calculated and normalized. The relationship between the normalized dispersion coefficient and the duty cycle is shown in Figure 13.

When the duty cycle is less than 50%, the normalized dispersion coefficient decreases rapidly with the increase of the duty cycle, as shown in Figure 13. We can conclude that the anti-interference ability of the system increases with the duty cycle in this case. When the duty cycle exceeds 50%, the decrease of normalized dispersion factor is not apparent, and raising the duty cycle can not improve the anti-interference ability of the system effectively. This phenomenon can be explained by energy. When the duty cycle increases, the light energy detected the photodetector increases accordingly. Therefore, the signal-to-noise ratio and anti-interference ability of the system increases with the light power. However, when the light intensity reaches a certain level, it is impossible to improve the signal-to-noise ratio and anti-interference ability by increasing the optical energy [21].

#### 4.2.2. The Effect of the Frequency Multiplication Factor

**Figure 14:**
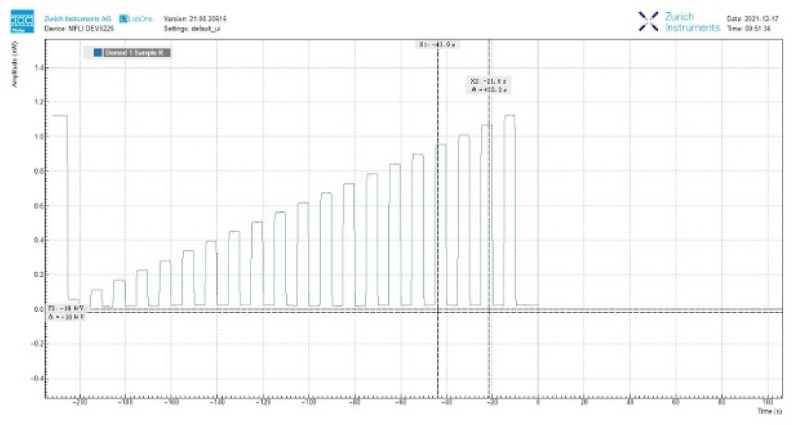
The software screenshot of the demodulation result with the lock-in amplifier.

We fixed the duty cycle and tested the impact of the frequency multiplication factor on the optical system. The duty cycle of AOM was 50%. We outputted the electric current with amplitude modulated by DC stabilized power supply. The software screenshot of the demodulation result of the lock-in amplifier is shown in Figure 14.

**Figure 15:**
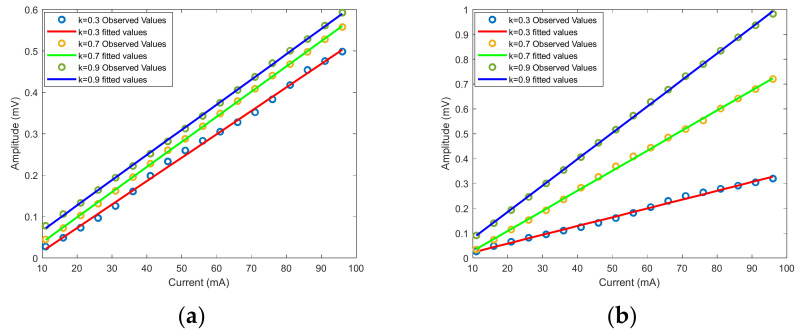
The observed values and fitted values with different frequency multiplication factors. (**a**) The duty cycle is fixed at 50%. (**b**) The duty cycle equals the frequency multiplication factors.

The frequency multiplication factor is 0.3, 0.7, and 0.9. The range of the electric current is 11 mA to 96 mA, and the interval is 5 mA. The measured data is filtered by the moving average filter and fitted by the polynomial fitting method. The observed and fitted values are shown in Figure 15.

The fitted value has good linearity with the measured value, as shown in Figure 15. The normalized scale factor and the ratio error were obtained by calculation, and the result is given in Table 2. 

The normalized scale factor is nearly the same when the duty cycle is fixed at 50%, as shown in Figure 15a and Table 2. Therefore, we can conclude that the duty cycle is the main factor that affects the normalized scale factor, which is consistent with the simulation in Section 3.2 and the experiment results in Section 4.2.1.

When the duty cycle of AOM equals frequency multiplication factors, and the frequency multiplication factor is an integer multiple of 0.1, the ratio error is small, as shown in Figure 15b and Table 2, which is consistent with the previous analysis in Section 3.1.

The sensitivity of AFOCS is the slope of the fitting curve, which is 0.01063 mV/mA when the duty cycle is 90%, and the frequency multiplication equals the duty cycle of AOM. The lock-in amplifier determines the resolution. The output range of the lock-in amplifier is ±10 V, and the D/A conversion is 18 bit [22]. The resolution of AFOCS is about 3 mA by computing. The measurement range of AFOCS is 11 mA to 196.62 A by calculation. 

## 5. Conclusions

Based on the above analysis, we can draw the following conclusions.

First, with an AOM and EOM, the AFOCS with a pulsed light source is realized. 

Second, the output of correlation demodulation is determined by the parameters of AOM. The duty cycle mainly affects the normalized scale factor, and the frequency multiplication factor nearly has nothing to do with the normalized scale factor.

Thirdly, the frequency multiplication factor is the link between AOM and EOM. The frequency multiplication factor mainly affects the ratio error. When the frequency multiplication factor equals the duty cycle, and both of them are integer multiple of 0.1, the system has good linearity and meets the microcurrent measurement requirements.

## Figures and Tables

**Table 1:** The experimental equipment and main parameters.

Equipment or Device	Enterprise or Brand	Model or Main Parameters
Light source	Thorlabs China Co., Ltd., Shanghai, China.	S5FC1018P.
Polarizer	MC Fiber Optics Co., Ltd., Shenzhen, China.	The extinction ratio is no less than 28 dB.
Circulator	MC Fiber Optics Co., Ltd., Shenzhen, China.	
EOM	SWT OPTICS Co., Ltd., Beijing, China	The half-wave voltage is 4 V.
Delay line	YOFC Optical Fiber and Cable Co., Ltd., Wuhan, China	PM1016-A.
Quarter-wave plate		Do it by ourselves.
Sensing fiber	YOFC Optical Fiber and Cable Co., Ltd., Wuhan, China.	SH-1016A, beat length is 10 mm, the spin pitch is 5 mm
Mirror	YOFC Optical Fiber and Cable Co., Ltd., Wuhan, China.	Reflectivity is greater than 99%@1310 nm.
Photodetector	Conquer Co., Ltd., Beijing, China.	KG-HSP.
Lock-in amplifier	Zurich Instruments China, Shanghai, China.	MFLI 500 kHz.
DC stabilized power supply	Rohde & Schwarz, Muenchen, Germany.	HMP4040.
Signal generator	Tektronix China Inc., Shanghai, China.	AFG 1062.

**Table 2:** The result of experiments with different frequency multiplication factors.

Duty Cycle	Frequency Multiplication Factors	Ratio Error	Normalized Scale Factor
50%	0.3	20.59%	0.5279
50%	0.7	5.24%	0.5710
50%	0.9	3.19%	0.5725
30%	0.3	9.27%	0.3309
70%	0.7	4.65%	0.7608
90%	0.9	1.79%	1

## References

[1] Blake J., Tantaswadi P., Carvalho R. (1996). In-line Sagnac interferometer current sensor. IEEE Trans. Power Deliv..

[2] Chen J., Xu Q., Wang K. (2020). Research and Application of Generator Protection Based on Fiber Optical Current Transformer. IEEE Access.

[3] Gubin V.P., Starostin N.I., Przhiyalkovsky Y.V., Morshnev S.K., Sazonov A.I. (2019). Recording of pulsed currents by a fibre-optic Faraday effect-based sensor with limited frequency band. Quantum Electron..

[4] Zhao J., Shi L., Sun X.H. (2021). Design and Performance Study of a Temperature Compensated ±1100-kV UHVDC All Fiber Current Transformer. IEEE Trans. Instrum. Meas..

[5] Gusarov A., Leysen W., Beaumont P., Wuilpart M., Dandu P., Boboc A., Croft D., Bekris N., Batistoni P. (2021). Performance assessment of plasma current measurement at JET using fibre optics current sensor. Fusion Eng. Des..

[6] Yang F.Q., Sun S.P., Sima W.X., He Y.X., Luo M.D. (2019). Progress of Advanced Voltage/Current Sensing Techniques for Smart Grid. High Volt. Eng..

[7] Burns W.K., Chen C.L., Moeller R.P. (1983). Fiber-Optic Gyroscopes with Broad-Band Sources. J. Light. Technol..

[8] Burns W., Moeller R. (1984). Polarizer Requirements for Fiber Gyroscopes with High-Birefringence Fiber and Broad-Band Sources. J. Lightwave Technol..

[9] Zhang H., Qiu Y., Li H., Huang A., Chen H., Li G. (2012). High-current-sensitivity all-fiber current sensor based on fiber loop architecture. Opt. Express.

[10] Liu Y., Chen J.B., Shu X.M., Hu W.P., Ding Q.Y., Ma Z.Y., Wei Z.B. (2008). Fiber Optical Current Transformers based on Modulated Light Source. J. Xi’an Jiao Tong Univ..

[11] Zhang H., Jiang J., Zhang Y., Chen H., Zhao N., Lin L., Qiu Y. (2017). A loop all-fiber current sensor based on single-polarization single-mode couplers. Sensors.

[12] Du J., Tao Y., Liu Y., Ma L., Zhang W., He Z. (2016). Highly sensitive and reconfigurable fiber optic current sensor by optical recirculating in a fiber loop. Opt. Express.

[13] Xin G., Zhu J., Luo C., Tang J., Li W., Cao Y., Xu H. (2020). Polarization Error Analysis of an All-Optical Fibre Small Current Sensor for Partial Discharge. J. Electr. Eng. Technol..

[14] Wu J., Zhang X., Chen L., Wu B. (2021). Research on Measurement Technology of Ship Leakage Current by All-Fiber Optic Current Sensor. IEEE Access.

[15] Mihailovic P., Petricevic S. (2021). Fiber Optic Sensors Based on the Faraday Effect. Sensors.

[16] Pang F.B., Liu Y., Yuan Y.B., Gao L. (2020). Influencing factors analysis on the detector output signal of fiber optic current transformer with sine modulation. Meas. J. Int. Meas. Confed..

[17] Cai W., Xing J.H., Yang Z.Y. (2017). Contributions to Verdet constant of magneto-optical materials. Wuli Xuebao/Acta Phys. Sin..

[18] Hu G.S. (2012). Digital Signal Processing Theory, Algorithm and Implementation.

[19] Lin W.G. (1955). Application of the Approximate Evaluation of Bessel Functions to Frequency Modulation System. Wuli Xuebao/Acta Phys..

[20] Gao J.Z. (2011). Detection of Weak Signals.

[21] Hu B., Xiao H., Li J.G., Wei S.P., Li H.B., Li Y.B., Wang X.Z., Liu D.W., Yang C.H. (2017). Noise Analysis and SNR Optimization Design of Fiber Optical Current Transforms. High Voltage Eng..

[22] https://www.zhinst.cn/china/en/products/mfli-lock-amplifier#multi-device-synchronization.

